# Association between breakthrough infection with COVID-19 and *Toxoplasma gondii*: a cross-sectional study

**DOI:** 10.1038/s41598-023-44616-3

**Published:** 2023-10-17

**Authors:** Marwa A. Gouda, Hind S. AboShabaan, Ahmed S. Abdelgawad, Aliaa Sabry Abdel Wahed, Khaled A. Abd El-Razik, Yara Elsaadawy, Ayman. A. Abdel-Wahab, Yousry Hawash

**Affiliations:** 1 Department of Clinical and Molecular Parasitology, National Liver Institute, Menoufia University, Shibin El Kom, Menoufia Egypt; 2 Department of Clinical Pathology, National Liver Institute, Menoufia University, Shibin El Kom, Menoufia Egypt; 3 Department of Hepatology and Gastroenterology, National Liver Institute, Menoufia University, Shibin El Kom, Menoufia Egypt; 4 Department of Animal Reproduction, National Research Centre (NRC), Dokki, Giza, Egypt; 5 Department of Medical Microbiology, Immunology, and Infection Control, Faculty of Medicine, Ain Shams University, Cairo, Egypt

**Keywords:** Clinical microbiology, Microbiology

## Abstract

The breakthrough infection following COVID-19 vaccination has been a subject of concern recently. Evidence suggests that COVID-19 vaccine efficacy diminishes over time due to multiple factors related to the host, and vaccine. Coinfection with other pathogens was claimed earlier as a contributing cause for this phenomenon. Hence, we aimed to stratify the association of post-COVID-19 vaccination breakthrough coinfection with *Toxoplasma gondii* (*T. gondii*) and its impact on disease severity. This cross-sectional study included 330 COVID-19-vaccinated patients confirmed by RT-PCR. They were also screened for anti- *T. gondii* antibodies using ELISA. *Toxoplasma* seropositive cases’ whole blood was screened for DNA using PCR to correlate results with COVID-19 severity. Out of 330 COVID-19 vaccinated patients with breakthrough infection, 34.5% (114 patients) showed positivity for *Toxoplasma* IgG by ELISA, and none of the cases was IgM positive. Eleven patients (9.6%) of the IgG-positive cases were positive by PCR. Positive PCR cases correlated positively with the *Toxoplasma* IgG titer (*P* < 0.001), and the Cutoff point was 191.5. Molecular analysis of *Toxoplasma* and COVID-19 severity showed that 8 (72.7%), 1 (9.1%), and 2 cases (18.2%) had mild, moderate, and severe courses of the disease, respectively, with no significant correlation. Our study reported a heightened prevalence of latent toxoplasmosis among mild cases of COVID-19 breakthrough infection. Nevertheless, a discernible correlation between latent toxoplasmosis and COVID-19 severity is lacking. Hence, implementing studies on a larger scale could provide a more comprehensive comprehension of this association.

## Introduction

Toxoplasmosis, brought on by the obligatory intracellular parasite *T. gondii,* is one of the most prevalent foodborne diseases concerning hospitalization and mortality, making it a serious global public health concern^1^. *Toxoplasma* is widely recognized as a highly adaptable protozoan organism; having the capacity to endure within host cells throughout the host's lifespan^2^.

Among different parasitic infections, toxoplasmosis is a suitable infection model to test the mutual influence of post-COVID-19 vaccine breakthrough coinfection with protozoa owing to two factors. First, it is global prevalence infecting around one-third of the global population. The Spread of this protozoan has several routes, including consuming raw or undercooked meat, ingesting sporulated oocytes excreted by cats, and receiving blood transfusions. However, it cannot be passed from person to person; its prevalence can be used as a general indicator of group cleanliness^2^. Second, this eukaryotic intracellular coccidian protozoan is recognized to have at least some antiviral effects^3^.

Infection with *T. gondii* may pass asymptomatic in healthy people; nevertheless it can be fatal in patients with weakened immune systems (AIDS, bone marrow transplant, and neoplasia) due to either the parasite's reactivation or a recent acute infection^4^. The severe lethal consequences of *Toxoplasma* infection in immunocompromised make it challenging to diagnose, which has been enhanced lately by integrating serology and molecular techniques. Some authors observed that high IgG anti-*T. gondii* antibodies titer is linked with positive PCR results in AIDS patients; others, likewise, demonstrated no correlation between IgG anti-*T. gondii* antibody titer and positive PCR among healthy blood donors^5,6^.

The global health emergency created by coronavirus (COVID-19), which was induced by the severe acute respiratory syndrome coronavirus-2 pathogen (SARS-CoV-2), caused the severe acute respiratory syndrome that killed Thousands of people^7^.

COVID-19 primarily immune response involves activating innate cells and virus-specific T cells and B cells; meanwhile, extensive inflammatory syndrome is characterized by the unregulated synthesis of pro-inflammatory cytokines and chemokines, and the progression of this condition can result in pulmonary injury, subsequently leading to respiratory failure and ultimately culminating in mortality^8,9^. Respiratory viral illnesses, in turn, increase the vulnerability of individuals to subsequent parasitic infections, as recognized in earlier research. These secondary infections typically result in a more unfavorable prognosis for the affected patients^10^.

With the swift and detrimental proliferation of COVID-19 contagion and the lack of targeted antiviral therapy, global cooperation became imperative to pursue vaccine development. Over five billion individuals globally were immunized with COVID-19 vaccines till 2022 end^11^. However, the trend of unexplained reduction in the effectiveness of commonly administered vaccines and the occurrence of breakthrough infections is a worrisome development in regions where diseases are prevalent. This trend also significantly challenges global efforts to control infections^6,12^.

The objective of this study was to examine the reciprocal relationship between COVID-19 vaccination and *Toxoplasma* infection, as well as” the subsequent influence of this association on the prevalence of *Toxoplasma* infection and COVID-19 severity by molecular diagnosis in Menoufia Governorate, Egypt. Moreover, we will investigate the theory that *T. gondii* parasitemia is correlated with high serum levels of anti-*T. gondii* antibodies titer.

## Methods

### Sample size calculation

According to Geraili et al.^13^, who found that the seroprevalence (IgG) of *T. gondii* among COVID-19 positive cases was 26.1%, at a marginal error of 0.05, the power of the study was 95%, the estimated sample size was a minimum number of 296 and after addition 10% (30 cases) for any suspected defect in the patient's records or lab analysis. The final minimal sample was 326 COVID-positive cases.

$$\frac{{{\text{n}} = {\text{Z}}_{\alpha - 1}^{2} * \, ({\text{pq}})}}{{{\text{e}}^{2} }}$$

The variable n represents the sample size, while the variable z denotes the standard error associated with the selected degree of confidence, which is 1.96., p = proportion detected in the reference study (26.1%), q = 1−p, and e = acceptable sample error (0.05).

### Study setting, site, and subjects

This cross-sectional observational study was conducted at various healthcare facilities, namely the ICU, hospitalized ward, outpatient care clinic, post-COVID-19 medical centers, and Vaccination Unit, The chest Division, of Menoufia University hospitals. The study was carried out from October 2021 with the start of ethical committee approval and ended with collection of calculated sample size. We recruited 330 patients vaccinated against COVID-19 in the study after getting their approval to participate. Recruited patients fulfilled the inclusion criteria of having concurrent coronavirus, screened positive by Reverse Transcription polymerase chain reaction (RT-PCR) in nasopharyngeal swabs, and *T. gondii* infection confirmed by ELISA. Patients with negative RT-PCR results and those with confirmed other acute illnesses were excluded from the study. Patient demographics, including age, gender, isolation location of cases, survival, and comorbidities such as hypertension, diabetes mellitus, and other chronic diseases, were available through a questionnaire, doctors' notes, and records of the medical exams, which provided pertinent information. All patients had thorough physical examinations, and the severity of COVID-19 was assessed following Egyptian MOH guidelines^14^. Mild cases with minor clinical signs, no dyspnea or shortness of breath, and normal chest imaging. Moderate cases called for intensive monitoring and home isolation. Moderate cases with lower respiratory infection symptoms and oxygen saturation (SpO_2_) of less than 92% in room air at sea level. They were received at the hospital in isolation. Severe cases that met any of the following requirements: During 24 to 48 h, the chest radiological findings had progressed by more than 50%, the respiratory rate was greater than 30 breaths per minute, and the oxygen saturation (SpO_2_) was less than 92%.

### Blood sampling and procedures

Each participant in the study donated six mL of venous blood collected from their cubital vein divided into two parts. Three ml were slowly transferred into an EDTA vacutainer tube frozen at − 20 °C for subsequent DNA extraction, and three ml were transferred into a plain tube, then centrifuged to separate the serum and stored at − 20 °C for subsequent biochemical analysis.

The immunoassay enzyme approach was employed to assess the seropositivity and the titer of serum antibodies against *T. gondii*. Using the manufacturer's recommended procedures, we used commercial kits to measure the serum concentration of IgG and IgM antibodies (Cat No. 201-12-1847, 201–-12-1845 Shanghai Sunred Biological Technology Co., Ltd).

Genomic DNA Extraction was carried out from EDTA samples using Gene JET Whole Blood Genomic DNA Purification Mini Kit according to the manufacturer's instructions (Catalog No K0721, Thermo Scientific). Positive controls (RH and ME 49) were gifted from the Zoonotic Diseases Department, National Research Centre (Cairo, Egypt), after DNA extraction for RH and ME 49 strains of *T. gondii*. The isolated DNA was kept at 20 °C.

**Figure 1:**
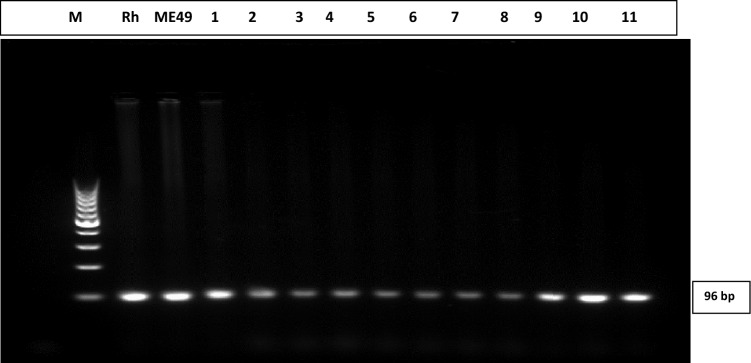
Agarose gel electrophoresis of PCR results. Agarose gel electrophoresis showing *T. gondii* DNA detection results by conventional PCR. From left to right the first two lanes correspond to positive control samples of Rh and ME49 strains respectively; Lanes 3–13 represent positive samples at 96 bp. M refers to Molecular band size. The image was taken using the gel documentation system directly.

The conventional PCR reaction was performed in a 25 μl total volume using five μl of genomic DNA, 0.5 μl of each primer, and 12.5 µl of master mix [Dream Taq Green PCR Master Mix (2X), Catalog no. K1081; Thermo Scientific] and 6.5 μl of H2O. The primers of the B1 gene were as follows: forward primer 5′-CGACAGCCGCGGTCATTCTC -3′ and reverse primer 5′-GCAACCAGTCAGCGTCGTCC 3′. were used according to the protocol previously applied. The length of the expected target is 96 bp. A ladder of 50 bp was used^15^ (Fig. 1).

### Statistical analysis

SPSS (statistical software for social science) version 20.0 was used on an IBM-compatible computer to gather and analyze the data (SPSS Inc., Chicago, IL, USA). (See “Diagnostic usefulness of GeneXpert MTB/RIF assay versus traditional…”) Spearman correlation was used to assess the relationship between IgG concentration and other quantitative parameters, and ROC curve analysis was performed. Qualitative data were described as frequency and percentage and analyzed using the chi-square test. In contrast, quantitative data were described as mean, standard deviation, and range. They were compared between 2 subgroups using the Mann–Whitney U test and between categories of comorbidities using the Kruskal–Wallis’s test. The *P* value was considered significant when < 0.05.

### Ethical approval

Research Ethics Committee from the Institute of National Liver Disease (NLI IRB protocol N. 00334/2022) approved this investigation. The research adhered to pertinent guidelines and regulations outlined in the Declaration of Helsinki.

### Consent to participate

Everyone who took part in this study was told of the study's goals and potential puncture side effects and provided their informed permission was obtained from all subjects and/or their legal guardian(s).

## Results

**Figure 2:**
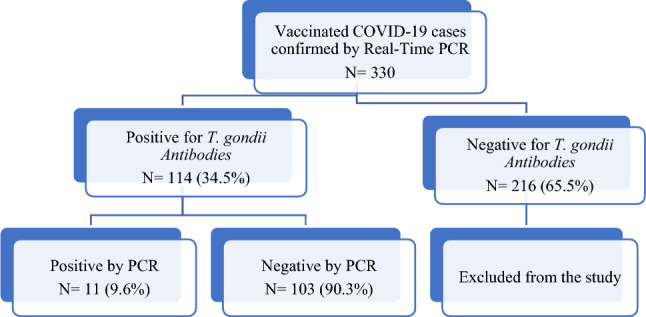
Flowchart of the participants in the study.

Three hundred thirty vaccinated patients against COVID-19 whose PCR tests had given the COVID-19 positive diagnosis were screened for *T. gondii* antibodies IgG & IgM. 114/330 showed positivity for *T. gondii* IgG by ELISA, and none of the screened cases was IgM positive. Only positive cases for the two selected parameters, COVID-19, and positive toxoplasmosis, were included in this study representing 34.5% of screened cases (114) (Fig. 2).

### Patients’ characteristics and outcome concerning the titer of *Toxoplasma* IgG

Table 1 lists patients’ characteristics and outcome**s** of the patients who took part in the trial. The patients ranged in age from 14 to 78, with a mean age of 47.69 years, 36.8% (42) of whom were men, and 63.2% (72) were women. 15.8% of patients had hypertension (HTN), 9.6% had diabetes mellites, respiratory diseases (asthma, chronic obstructive pulmonary disease (COPD) and others), and heart disease was reported at 1.8 & 4.4%, respectively, and 68.4% of those patients were without comorbidities. COVID-19 had a modest severity; nonetheless, 17.5% of patients needed hospitalization due to a severe condition, and mortality was recorded as 2.6%.

**Table 1:** Patients' characteristics and outcome concerning the titer of *Toxoplasma* IgG.

	IgG titre among the studied cases N = 114		U test	P value
	Mean ± SD	Range		
Sex				
Male (42)	77.14 ± 89.41	13.5–340	0.55	0.58
Female (72)	94.90 ± 102.05	10.4–350		
Comorbidities			K	
Negative (78)	84.96 ± 96.15	11.8–350	4.62	0.33
HTN (18)	82.61 ± 89.72	13.5–320		
DM (11)	117.09 ± 110.01	12.0–340		
Asthma& COPD (2)	140.2 ± 126.99	50.4–230		
Cardiac (5)	78.26 ± 135.34	10.4–320		
Site of isolation			U	
Home (94)	82.83 ± 91.19	10.4–350	0.43	0.67
Hospital (20)	114.37 ± 122.63	11.8–340		
Outcome			U	
Survived (111)	87.3 ± 95.49	10.4–350	0.74	0.75
Died (3)	127.43 ± 184.25	13.5–340		

	r		*P* value	
Age	0.05		0.57	

U = Mann Whitney U test, K = Kruskal Wallis test.

### Demographic and clinical parameters of positive *Toxoplasma* cases by PCR

One hundred and fourteen COVID-19 patients with positive toxoplasmosis results were assessed for B1 gene detection using PCR. Eleven patients out of the 114 *Toxoplasma* IgG-positive cases had positive PCR results; eight were women, and three were men. (Table 2). The results showed no significant difference in the mean age of participants and gender regarding PCR positivity (*P* > 0.05). 9.1% of positive cases had HTN. Respiratory and heart diseases were reported at a rate of 9.1% for each comorbidity, and 27.7% of those patients were without comorbidities. Eight cases had mild COVID-19 severity at 72.7%, while two needed hospital admission due to a severe condition, and mortality was recorded as 9.1% with no significant difference regarding severity recorded.

**Table 2:** Demographic and clinical parameters among positive *Toxoplasma* cases in relation to PCR results.

Variables	PCR positive N = 11	PCR Negative N = 103	Test	*P* value
Age				
Mean ± SD	49.91 ± 18.27	47.45 ± 16.33	U	0.71
range	27–75	14–85	0.37	
Age				
≤ 40	4 (36.4)	42 (40.8)	X^2^	0.51
41–60	3 (27.3)	39 (37.9)	1.33	
> 60	4 (36.4)	22 (21.4)		
Gender				
Male	3 (27.3)	39 (37.9)	FE	0.74
Female	8 (72.7)	64 (62.1)	0.48	
Comorbidities				
Negative	8 (27.7)	70 (68.0)		
HTN	1 (9.1)	17 (16.5)	X^2^	0.21
DM	0 (0.0)	11 (10.7)	5.9	
Respiratory diseases	1 (9.1)	1 (1.0)		
Cardiac	1 (9.1)	4 (3.9)		
Site of cases isolation				
Home	7 (63.6)	87 (84.5)	FE	0.10
Hospital	4 (36.4)	16 (15.5)	2.98	
Severity of COVID-19				
Mild	8 (72.7)	60 (58.3)	X^2^	0.42
Moderate	1 (9.1)	28 (27.2)	1.71	
Sever	2 (18.2)	15 (14.6)		
Outcome				
Survived	10 (90.9)	101 (98.1)	FE	0.26
Died	1 (9.1)	2 (1.9)	1.98	

U = Mann Whitney U test, X^2^ = Chi square test, FE = Fisher's Exact test.

### Association between the ELISA concentration of *Toxoplasma* IgG antibodies and PCR

**Figure 3:**
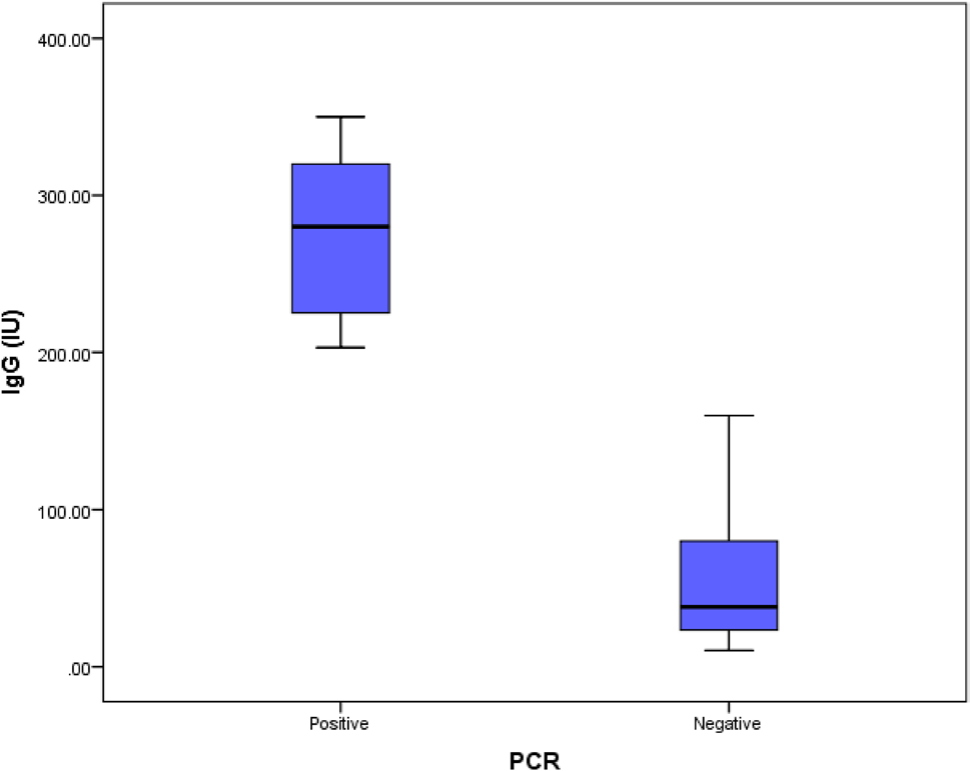
PCR findings in relation to the concentration of *Toxoplasma* IgG antibodies.

*T. gondii's* B1 gene underwent conventional PCR amplification. The results revealed a 9.6% positive rate among IgG seropositive cases. Anti-*Toxoplasma* IgG antibody concentration was significantly higher value in PCR positive group than those with negative PCR results (274.0 ± 53.69 vs. 68.54 ± 78.41), respectively (*P* < 0.001) (Table 3). (Fig. 3).

**Table 3:** PCR findings in relation to the concentration of *Toxoplasma* IgG antibodies.

	Seropositive COVID-19 patients N = 114		U test	*P* value
	Positive PCR N = 11	Negative PCR N = 103		
IgG				
Mean ± SD.	274.0 ± 53.69	68.54 ± 78.41	4.85	< 0.001*
Range	203–350	10.4–350		

*Significant *P* value < 0.001.

**Figure 4:**
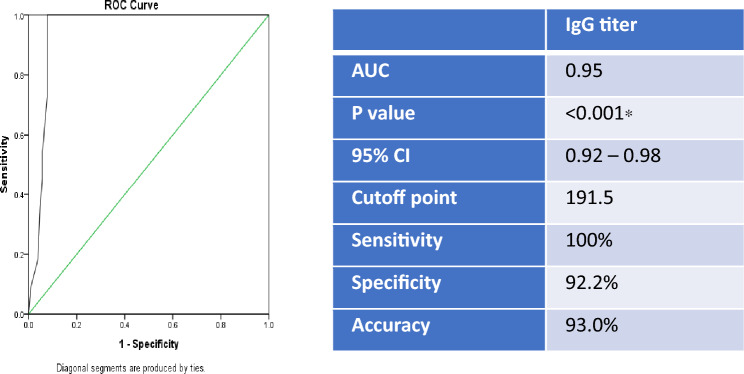
ROC Curve analysis for *Toxoplasma* IgG titer in predicting PCR positivity.

We used ROC curve in current work to assess the sensitivities and specificities for various values. The best cut-off was determined to be an ELISA IgG abs concentration of 191.5 IU/mL, with diagnostic specificity, sensitivity, and accuracy being 92.2%, 100%, and 93.0%, respectively. ROC analysis showed an area underneath the curve (AUC) of 0.95 in relation to the IgG levels (Fig. 4).

## Discussion

The present study recorded chronic infection with toxoplasmosis among 34.5% of patients with coronavirus breakthrough infection following vaccination with the COVID-19 vaccine. At the same time, no active cases were recorded by ELISA. Humans are frequently co-infected with viruses and protozoa. The immunosuppression effects of infections with protozoa can reduce the effectiveness of immunizations, decrease the establishment of immunological memory, and reduce protection against co-infecting pathogens^12^.

In other earlier studies, the seroprevalence of *T. gondii* among COVID-19 patients was recorded in Egypt, where the prevalence was 22.4%^16^. In Iran, Ghaffari et al.^6^, found that 84% of the patients with COVID-19 had anti-*T. gondii* antibodies (IgG). In contrast Geraili et al.^13^, reported a 26.1% prevalence rate in Northern Iran. This variation in toxoplasmosis prevalence between studies may have been influenced by local environmental factors such as humidity, geography, and age distribution of the research population. According to earlier reports, infected people are more likely to get toxoplasmosis in humid and warmer climates^17^. We also refer to the variation of the infection of toxoplasmosis reported before vaccine development to the restriction system applied by different countries to coronavirus.

This work found *T. gondii* DNA in eleven cases with positive IgG and negative IgM antibodies and high IgG titers by conventional PCR amplification of the B1 gene, constituting 9.6% of the positive results by ELISA. Fallahi et al.^18^ involved children with cancer in their study. They found that molecular studies provide superior sensitivity and specificity for diagnosis, especially using B1 genes utilized often in the *T. gondii* DNA detection. Ibrahim et al.^19^ also determined the prevalence based on B1 amplification in Egypt with similar results.

One of the main insights of the current work was the significant correlation between *Toxoplasma* antibodies titer and PCR results where *T. gondii* positive cases were correlated with higher antibodies IgG titer in serum with a significant difference. To our knowledge, this is the first study in Egypt to correlate *T. gondii* parasitemia in COVID-19 breakthrough infection following vaccination using a molecular approach. This finding is supported by Halleyantoro et al.^20^, who found that positive PCR samples had greater anti-*Toxoplasma* IgG titer in HIV encephalitis patients in Iran using the B1 gene. Also, nine women with high IgG titer of ≥ 200 IU had *T. gondii* DNA in their blood samples, indicating that they may have contracted a recent infection^21^. The data presented here are not supported by other reports that observed a significant linkage between toxoplasmosis and IgM anti-*T. gondii* antibodies, despite reporting the potentiality of infection to IgG antibodies carriers^20,21^.

SARS-CoV-2 severity and mortality are impacted by innate and adaptive immune responses to two primary immune dysregulations. One is cytokine storm with overproduction of pro-inflammatory cytokines, which leads to immune exhaustion, which was also reported experimentally following vaccination^22^. The other is a dysregulation of lymphocytes that leads to B-cell lymphopenia and is characterized by CD4 + T cells^23^. Due to the cellular immune system's deficiency in these individuals, the infection by *T. gondii* may be reactivated. The parasite may be found in the blood as tachyzoites, revealing positive cases by the molecular test. In the latest study, CD8 T dysfunction brought on by CD4 T cell depletion was considered a contributing factor to the infection's reactivation in persistently infected hosts^24^. Subjects infected with *Toxoplasma* were more likely to develop Covid-19 and have a more severe disease course; they were also more likely to require hospitalization and intensive care unit treatment^25^.

The Association between *Toxoplasma* DNA and COVID severity post-vaccine was explored in the present work. Mild cases were more associated with the *Toxoplasma* DNA-positive cases; however, the association was non-significant. Hamad & Garedaghi^26^ hypothesized that the influenza virus parasitizes *T. gondii.* This symbiotic relationship lessens *T. gondii's* pathogenicity while increasing the survival of the influenza virus. As a result, patients experience the flu longer than is typical. In contrast, others found that toxoplasmosis is prevalent in COVID patients as a part of the general population and is not considered a risk factor for disease severity^13^.

Considering recent studies of cases of breakthrough infection after the COVID vaccine the reported data about disease severity recorded 27% of asymptomatic cases, with the need for hospitalization in ten percent; it seems that toxoplasmosis in our research affected the severity of disease and death rate recorded^27^. However, other factors may be responsible for mild cases observed; one important factor is the emergence of the Omicron variant, characterized by its unparalleled transmission rate, heightened immune evasion, transmissibility, and reduced pathogenicity^28,29^. Breakthrough infections could be attributed to other factors, including characteristics of immunity, host determinants (such as age, comorbidities, immune status, and use of immunosuppressive drugs^30^. Hypertension, respiratory, and cardiac diseases exhibited a prevalence of comorbidity, with a rate of 9.1% for each. Our present study recorded no significant difference regarding different comorbidities and disease severity or coinfection which is in agreement with Montazeri et al.^31^.

Investigators considered the age of participants in the epidemiology of toxoplasmosis and correlated it with acquiring infection. They found that infection increases with age, reaching a peak in patients older than 50^32,33^. The COVID-19 patients' mean age (47.69 ± 16.53years) was lower than in the research listed above and similar to some extent to Abdel-Hamed et al.^16^ with a mean age of 44.72 ± 13.82years. Ghaffari et al.^6^ found that aging is not significantly associated with the prevalence of *Toxoplasma* Additionally, no correlation was found between gender and the prevalence of latent toxoplasmosis. These findings contradict earlier research that found a statistically significant relationship between *Toxoplasma* seropositivity and age, gender, and other factors^34^. Different study populations and study regions could be the cause of this discrepancy.

We know the drawbacks of small numbers of positive *T. gondii* DNA cases for concurrent infection with COVID and the difficulties of drawing any added conclusions about the relationships using molecular analysis. However, the sample size was calculated before the study. Nevertheless, because the current work demonstrates the effectiveness of molecular diagnostics and its correlation with serology titer, our data constitute a breakthrough in this research point. Its connection to ELISA will forecast COVID-19-potentially reactivated toxoplasmosis cases that should be considered in managing and relation to disease severity. The genotyping of *T. gondii* and the correlation between the severity of COVID will be considered a future next step.

## Conclusion and recommendations

The current investigation represents an initial endeavor to examine the frequency of chronic *T. gondii* infection in individuals who have experienced breakthrough infection of SARS-CoV-2 after vaccination. The findings indicate a high prevalence of latent *Toxoplasma* infection among individuals infected with SARS-CoV-2. Moreover, there was no discernible correlation found between COVID-19 and latent toxoplasmosis. Additionally, our findings indicate the need for future investigations to detect the impact of acute to chronic protozoan infection on immune-mediated changes and vaccine efficacy in individuals coinfected with both pathogens.

### Supplementary Information

Supplementary Information.

## Data Availability

The datasets utilized and examined in the present investigation can be obtained from the corresponding author upon a reasonable request.

## Abbreviations

AUC: The area under the ROC curve
Bp: Base pair
COPD: Chronic obstructive pulmonary diseases
COVID-19: Coronavirus disease 2019
DM: Diabetes mellitus
DNA: Deoxyribonucleic acid
EDTA: Ethylene diamine-tetra-acetic acid
ELISA: Enzyme-linked immunosorbent assay
HIV: Human immunodeficiency virus
HTN: Hypertension
IFA: Immunofluorescence antibody assay techniques
IgG: Immunoglobulin G
IgM: Immunoglobulin M
MOH: Egyptian Ministry of Health’s
PB: Peripheral blood
PCR: Polymerase chain reaction
RT-PCR: Reverse Transcription polymerase chain reaction
SARS-CoV-2: Severe acute respiratory syndrome coronavirus 2
SPSS: Statistical package for social science
*T. gondii*: *Toxoplasma gondii*
