# Supplementary material for: Association between breakthrough infection with COVID-19 and Toxoplasma gondii: a cross-sectional study
Source: Sci Rep. 2023 Oct 17;13:17636. doi: 10.1038/s41598-023-44616-3 (PMC10582182; doi:10.1038/s41598-023-44616-3)
Supplement: Supplementary file 1 — Supplementary Information. [file 41598_2023_44616_MOESM1_ESM.docx]

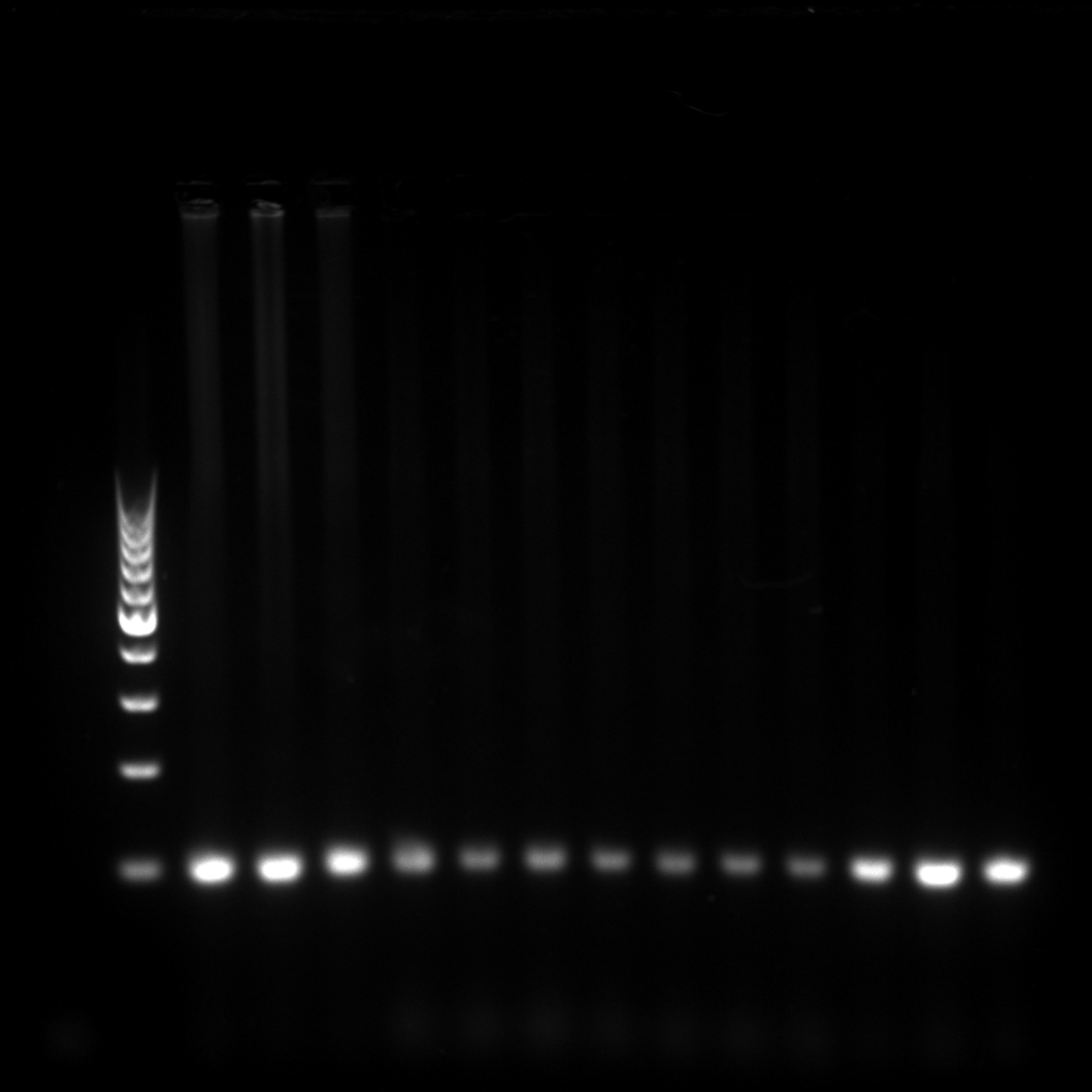


**96 bp**

**M Rh ME49 1 2 3 4 5 6 7 8 9 10 11**

**Fig.1** Agarose gel electrophoresis showing T. gondii DNA detection results by conventional PCR. From left to right the first two lanes correspond to positive control samples of Rh and ME49 strains respectively; Lanes 1-11 represent positive samples at 96 bp. The image was taken using the gel documentation system directly.
